# IFN-γ, IL-17A, IL-4, and IL-13: Potential Biomarkers for Prediction of the Effectiveness of Biologics in Psoriasis Patients

**DOI:** 10.3390/biomedicines12051115

**Published:** 2024-05-17

**Authors:** Ching-Liang Hsieh, Sheng-Jie Yu, Kuo-Lung Lai, Wei-Ting Chao, Chung-Yang Yen

**Affiliations:** 1 Chinese Medicine Research Center, China Medical University, Taichung City 404, Taiwan; clhsieh0826@gmail.com; 2 Department of Chinese Medicine, China Medical University Hospital, Taichung City 404, Taiwan; 3 Department of Medical Research, Taichung Veterans General Hospital, Taichung City 407, Taiwan; shengjieyu@vghtc.gov.tw; 4 Division of Allergy, Immunology and Rheumatology, Department of Internal Medicine, Taichung Veterans General Hospital, Taichung City 407, Taiwan; kllaichiayi@yahoo.com.tw; 5 Department of Life Science, Tunghai University, Taichung City 407, Taiwan; wtchao@thu.edu.tw; 6 Department of Dermatology, Taichung Veterans General Hospital, Taichung City 407, Taiwan; 7 School of Medicine, National Yang Ming Chiao Tung University, Taipei 112, Taiwan; 8 Integrated Care Center of Psoriatic Disease, Taichung Veterans General Hospital, Taichung City 407, Taiwan

**Keywords:** psoriasis, biologics, biomarker

## Abstract

Biologics are widely used to treat moderate-to-severe psoriasis. However, we have unmet needs for predicting individual patient responses to biologics before starting psoriasis treatment. We investigate a reliable platform and biomarkers for predicting individual patient responses to biologics. In a cohort study between 2018 and 2023 from a referral center in Taiwan, twenty psoriasis patients with or without psoriatic arthritis who had ever experienced two or more biologics were enrolled. Peripheral blood mononuclear cells obtained from these patients were treated with *Streptococcus pyogenes* and different biologics. The PASI reduction rate was strongly correlated with the reduction rate in the IL-13 level (*p* = 0.001) and the ratios of IFN-γ to IL-13 (*p* < 0.001), IFN-γ to IL-4 (*p* = 0.019), and IL-17A to IL-13 (*p* = 0.001). The PASI reduction difference was strongly correlated with the difference in the IFN-γ level (*p* = 0.002), the difference in the ratios of IFN-γ to IL-4 (*p* = 0.041), the difference in the ratios of IFN-γ to IL-13 (*p* = 0.006), the difference in the ratios of IL-17A to IL-4 (*p* = 0.011), and the difference in the ratios of IL-17A to IL-13 (*p* = 0.029). The biomarkers IFN-γ, IL-13, IFN-γ/IL4, IFN-γ/IL13, IL-17A/IL-4, and IL-17A/IL-13 are representative of the effectiveness of psoriasis treatment.

## 1. Introduction

Psoriasis is an autoimmune skin inflammation disease with a prevalence between 0.14% and 5.32% worldwide [1,2]. In recent decades, psoriasis treatment has substantially improved with the introduction of several new biologics [3,4]. Psoriasis Area Severity Index (PASI) scores can be reduced by 90% from baseline (PASI 90) or 100% from baseline (PASI 100) with the use of biologics. The estimated PASI 90 response rates at 44 to 60 weeks after beginning treatment were 79% for risankizumab, 76.5% for guselkumab, 74.0% for brodalumab, 73.9% for ixekizumab, 71.3% for secukinumab, 52.4% for ustekinumab, 46.2% for adalimumab, 40.1% for infliximab, 33.4% for etanercept, and 16.0% for apremilast [5]. These overall response rates, however, do not represent the real responses of individual patients to each biological agent. More information about selecting biologics was investigated, such as difficult-to-treat psoriasis areas, short-term effectiveness, and long-term drug survival on IL-17 and IL-23 inhibitors in biologics-naïve patients or after adalimumab failure [6,7,8]. Biologics-naive patients weighing ≦ 65 kg could be considered potential candidates for a half dose of risankizumab in treating psoriasis [9]. Additionally, crucial factors influencing an individual patient’s clinical response include the patient’s genetics, environmental factors, and autoantibody profile, as well as whether the patient is biologics-naïve [10,11,12]. In 2015, Boehncke et al. mentioned a need for a reliable platform for predicting individual patient responses to biologics before starting psoriasis treatment [13].

The medical treatment of psoriasis and psoriatic arthritis incurs substantial costs every year. Some patients do not respond favorably to a first clinical treatment with biological agents. These patients must then change to another biological agent to continue treatment. Due to the lack of a pre-administration evaluation and a screening platform, patients are often prescribed biological agents according to standard treatment guidelines instead of according to their individual cases. An improper selection of biologics causes the patient physical discomfort and psychological stress, as well as wasting health care resources.

A prescreen is the first step toward personal, precise treatment. Several studies investigated genes, including HLA-related and non-HLA-related single-nucleotide polymorphisms, tissue markers, and serum markers [14]. An HLA-C*06:02 negative genotype was more likely to respond to adalimumab than ustekinumab [15]. Conversely, patients achieved a PASI75 outcome with ustekinumab; 92% of HLA-C*06:02 positive patients achieved this compared with 67% of HLA-C*06:02 negative patients [16]. Several SNPs were identified as potential biomarkers in response to tumor necrosis factor (TNF)-α inhibitor biologics [17]. In recent studies, tissue and serum markers seem to be controversial [18]. So far, there is no useful or reliable platform or biomarker that can predict the effectiveness of biologics before starting treatment.

Peripheral blood mononuclear cells (PBMCs), which are composed of lymphocytes, macrophages, dendritic cells, and basophils, are widely used for immune mechanism surveys and drug selection. Each individual’s PBMCs present that individual’s immune function and characteristics. Therefore, PBMCs are useful in designing an individualized platform for screening the efficacy of biologics before starting treatment. *Streptococcus pyogenes* (*S. pyogenes*) can trigger immune responses to activate psoriasis outbreaks [19,20]. Furthermore, the innate immune system has been shown to be activated by *S. pyogenes* in both guttate and chronic plaque psoriasis [21]. The specific IgA response against *S. pyogenes* was correlated with a cutaneous lymphocyte-associated antigen+ T cell-dependent IL-17F response [22]. In this study, we use *S. pyogenes* for PBMC induction to simulate a real clinical psoriasis outbreak and treat it with different biologics. We survey different biomarkers that can represent clinical skin change in terms of the PASI score.

## 2. Materials and Methods

### 2.1. Participants

Our criteria for participating in this study were patients with psoriasis aged from 20 to 65 years old who had experienced at least 2 biological agents. We excluded patients with active infections or potential malignancies. A total of 20 patients with psoriasis with or without psoriatic arthritis were enrolled in this study from 2018 to 2023. All participants were from the clinic of either the Department of Allergy, Immunology, and Rheumatology or the Department of Dermatology at Taichung Veterans General Hospital. All participants provided written informed consent, and the study protocol was approved by the Institutional Review Board of Taichung Veterans General Hospital (TCVGH-CE16265B; TCVGH-CE20043B). The PASI score was accessed before and after the biologic treatments. We recorded the PASI score on day 0 and the PASI score closest to the time of PBMC testing on experienced biologics (at least 180 days) or the PASI score on day 180 for inexperienced biologics or experienced biologics (within 180 days).

### 2.2. Materials

*S. pyogenes* group A was identified and prepared by the Department of Pathology and Laboratory Medicine of Taichung Veterans General Hospital. *S. pyogenes* group A was inactivated by heat and then placed on blood agar plates for 1 week.

### 2.3. Cell Culture

To prepare the PBMC culture, 16 mL of blood was obtained from each patient and collected in sodium citrate tubes (Vacutainer CPT, BD Biosciences, Franklin Lakes, NJ, USA). Then, PBMCs were purified using centrifugation at 1800× *g* for 20 min at room temperature and with the brake off to generate distinct plasma, PBMCs, gel plugs, and red blood cell (RBC) layers. The cells were washed with phosphate-buffered saline and subsequently cultured in RPMI-1640 supplemented with 10% fetal bovine serum and 1% penicillin/streptomycin at 37 °C and 5% CO_2_.

### 2.4. Cell Viability Test

Patient PBMCs were cultured with 5 × 10^6^, 1 × 10^7^, and 2 × 10^7^ CFU concentrations of *S. pyogenes* for 24 h, and then 0.5 mg/mL of 3-(4,5-dimethylthiazol-2-yl)-2,5-diphenyltetrazolium bromide was added. After reacting for 1 h, the mixtures were centrifuged, and the supernatants were removed. Subsequently, 200 μL of dimethyl sulfoxide was added to lyse the cells and dissolve purple formazan crystals. We analyzed cell viability with an enzyme-linked immunosorbent assay reader at a wavelength of 570 nm.

### 2.5. Mutiplex Assay for Cytokine Levels

A total of 6 × 10^5^ cells per milliliter were then cultured in a 12-well plate and treated for 24 h with the following biologics: control, *S. pyogenes* only, *S. pyogenes* + adalimumab (4 μg/mL), *S. pyogenes* + golimumab (0.5 μg/mL), *S. pyogenes* + certolizumab (20 μg/mL), *S. pyogenes* + ustekinumab (0.25 μg/mL), *S. pyogenes* + ixekizumab (3.5 μg/mL), *S. pyogenes* + secukinumab (16.7 μg/mL), *S. pyogenes* + secukinumab (34 μg/mL), *S. pyogenes* + guselkumab (1.2 μg/mL), or *S. pyogenes* + risankizumab (2 μg/mL). Supernatants were collected for the subsequent measurement of cytokine levels. The concentrations of the biological agents we tested are the trough serum concentrations at a steady state indicated in the pharmacokinetic section of the reference list. The two concentrations of secukinumab (16.7 or 34 μg/mL) reflect the two common clinical doses of 150 mg or 300 mg per month, respectively.

To measure cytokine levels, culture supernatants were collected, and the concentrations of IL-1β, IL-2, IL-4, IL-5, IL-6, IL-7, IL-8, IL-10, IL-12, IL-13, IL-17A, IFN-γ, TNF-α, monocyte chemoattractant protein (MCP)-1, macrophage inflammatory protein (MIP)-1α, MIP-1β, platelet-derived growth factor-BB, and chemokine (CC motif) ligand 5 (RANTES) were determined using a protein multiplex immunoassay system (Bio-Plex Cytokine Array System, Bio-Rad Laboratories, Hercules, CA, USA).

### 2.6. Statistical Analysis

All statistical analyses were performed using SPSS version 22 (IBM, Armonk, NY, USA). Cytokine and chemokine levels in psoriasis patients and psoriatic arthritis patients were analyzed using the Mann–Whitney U test. Cytokine expression and PASI were analyzed with Spearman’s rho. Data were presented as the mean ± standard deviation. Two-sided *p* values of 0.05 or less were considered to indicate statistical significance.

## 3. Results

### 3.1. Cell Viability Test

**Figure 1:**
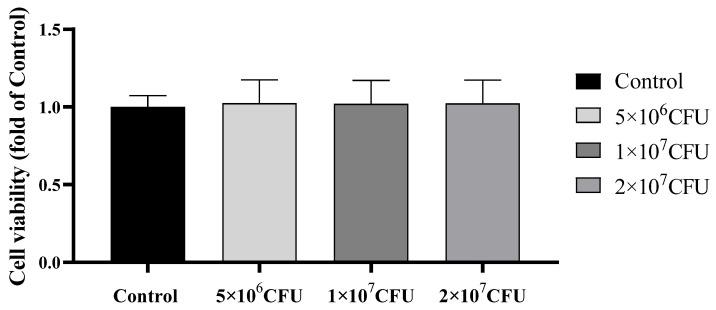
PBMCs isolated from psoriasis patients (*n* = 5) incubated with 5 × 10^6^, 1 × 10^7^, and 2 × 10^7^ CFU *S. pyogenes* for 24 h. Cell viability was measured by an MTT assay.

Five patients’ PBMCs were cultured with 5 × 10^6^, 1 × 10^7^, and 2 × 10^7^ CFU concentrations of *S. pyogenes* for 24 h. Similar cell viability to the control was recorded for the concentration of 5 × 10^6^, 1 × 10^7^, and 2 × 10^7^ CFU/mL. Next, 2 × 10^7^ CFU/mL of *S. pyogenes* group A was selected for similar cell viability and proper induction response (Figure 1).

### 3.2. Clinical Association between Clinical PASI and Laboratory Profile by Reduction Rate and Difference

Twenty patients were enrolled in our study. The demographic, time of PBMC testing, order of biologic sequences, and clinical characteristics of the psoriasis patients are listed in Table 1. The cytokine analyses of patients’ PBMCs and PASI scores are listed in Table 2. In this preliminary pilot study, the PASI reduction rate was strongly correlated with the reduction rate in the IL-13 level (*p* = 0.001) and the ratios of IFN-γ to IL-13 (*p* < 0.001), IFN-γ to IL-4 (*p* = 0.019), and IL-17A to IL-13 (*p* = 0.001). The PASI reduction difference was strongly correlated with the difference in the IFN-γ level (*p* = 0.002), the difference in the ratios of IFN-γ to IL-4 (*p* = 0.041), the difference in the ratios of IFN-γ to IL-13 (*p* = 0.006), the difference in the ratios of IL-17A to IL-4 (*p* = 0.011), and the difference in the ratios of IL-17A to IL-13 (*p* = 0.029; Table 3).

The PASI results represent the mean relative PASI. The results of PASI and consumption time were parallel to previous excel columns for different biologics.

In every patient, we showed the absolute PASI score before (day 0) and after biologic treatment. Laboratory profiles showed induction after *S. pyogenes* and treatment with biologics in different markers. Patient I received secukinumab 150 mg monthly, and patients E and G received secukinumab 300 mg monthly. The patients’ PBMCs were treated with secukinumab at 16.7 and 34 µg per milliliter, respectively.

The reduction rate of IFN-γ/IL-13 was calculated after treatment with biologics as compared with *S. pyogenes* induction only as follows:

(1) $$\frac{\left(\mathrm{IFN}-\gamma /\mathrm{IL}-13\;w/S.\;\;pyogenes\;\&\;\mathrm{biologics}\right)-\left(\mathrm{IFN}-\gamma /\mathrm{IL}-13\;w/S.\;\;pyogenes\;\mathrm{only}\right)}{\left(\mathrm{IFN}-\gamma /\mathrm{IL}-13\;w/S.\;\;pyogenes\;\mathrm{only}\right)}$$

* The difference in IFN-γ/IL-13 was calculated after treatment with biologics compared with *S. pyogenes* induction only as follows:

(IFN-γ/IL-13 w/*S. pyogenes* and biologics) − (IFN-γ/IL-13 w/*S. pyogenes* only) (2)

There is the same rationale for IFN-γ/IL4 and IL-17A/IL13 as above. 

### 3.3. Correlation between Clinical PASI and Biomarkers among Different Biologics

**Figure 2:**
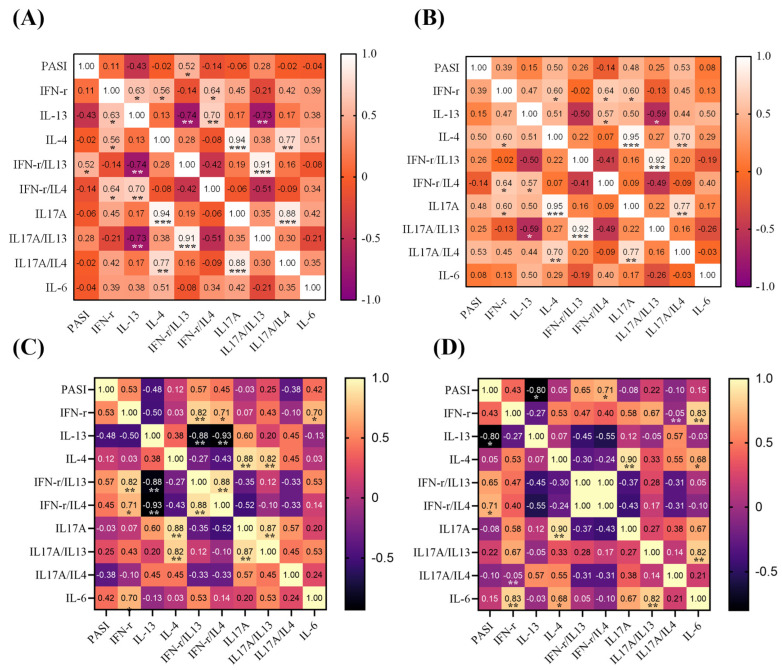
Correlation between clinical PASI and biomarkers among different biologics. Heatmaps showing correlation coefficients with different colors and *p* values with stars. (**A**) Reduction ratios of PASI and biomarkers for ustekinumab. (**B**) Reduction difference of PASI and biomarkers for ustekinumab. (**C**) Reduction ratios of PASI and biomarkers for adalimumab. (**D**) Reduction difference of PASI and biomarkers for adalimumab. * *p* < 0.05, ** *p* < 0.01, *** *p* < 0.001.

The level of IL-6 was weakly correlated with the PASI score reduction but was not significant. The levels of IL-1β, IL-2, IL-5, IL-7, IL-8, IL-10, IL-12, TNF-α, MCP-1, MIP-1α, MIP-1β, platelet-derived growth factor-BB, and RANTES were not correlated. The relation of biomarkers in different subgroup biologics was that the reduction ratios of IFN-γ to IL-13 were dominant with the PASI reduction difference in ustekinumab (*p* = 0.047; Figure 2A); the difference in the IL-17A level and the ratio of IL-17A to IL4 were correlated with the PASI difference but were not significant in ustekinumab (*p* = 0.068, *p* = 0.052; Figure 2B). The difference in the IL-13 level and the ratio of IFN-γ to IL4 were dominant with the PASI difference in adalimumab (*p* = 0.01, *p* = 0.047; Figure 2D). There was a lack of significance for ixekizumab, risankizumab, and guselkumab.

### 3.4. Correlation between Clinical PASI and Biomarkers in PsO Only and PsO + PsA Groups

We further investigated biomarkers in the PsO only and PsO + PsA subgroups. Similar biomarkers, including IFN-r, IL-13, IFN-r/IL-4, IFN-r/IL-13, IL-17A/IL-4, and IL-17A/IL-13, were significantly associated with clinical PASI, despite fewer samples in each group (Table 4).

## 4. Discussion

Psoriasis is a Th1/Th17-predominant disease. IFN-γ is a crucial cytokine in the pathogenesis of psoriasis. IFN-γ-producing Th1 lymphocytes can cause neutrophil infiltration, vessel proliferation, and keratinocyte hyperproliferation [23]. Kryczek et al. found that IFN-γ is a potent promoter of human IL-17+ T cell function, induction, and trafficking [24]. IFN-γ synergizing with IL-17 can enhance β-defesin-2 secretion, suggesting that Th1 and Th17 cells can cooperate to contribute to the pathogenesis of psoriasis [25]. IFN-γ injections in the unaffected skin of a patient with psoriasis can cause pathogenic T cell infiltration, dendritic cell infiltration, and IL-23 secretion [26]. Therefore, IFN-γ is a major cytokine in Th1 and plays a pivotal role in the Th17 pathway. In our pilot research, IFN-γ level was a primary marker reflecting the outcome of biologics before starting treatment. The Th17 pathway is another major effector cytokine in the pathogenesis of psoriatic disease [27]. The relationship between the ratio of IL-17A to IL-13 and PASI was also statistically significant. The ratio of IL-17A to IL-13 seems to be another potential marker. Th17/1 cells, which originate from Th17, will be activated in the chronic phase, re-challenged with stress, and then IL-17A and IFN-γ will be secreted to exacerbate clinical disease [28]. Taken together, IFN-γ as well as IL-17A are important markers in our research.

Our results suggest that psoriasis is not only associated with Th1 and Th17 but also Th2. An imbalance of Th1 and Th2 tends to exacerbate psoriasis symptoms. In 2003, Ghoreschi et al. developed an innovative therapy where IFN-γ-producing Th1 cells were selectively skewed toward IL-4-producing Th2 cells. Rather than suppressing the immune system, the researchers attempted to achieve immune balance by using IL-4 and found improvements in psoriasis treatment outcomes [29]. IL-4 and IL-13 are major cytokines in the Th2 reaction. In the present study, we analyzed the ratios of IFN-γ to IL-4, IFN-γ to IL-13, and IL-17A to IL-13, which were all significantly correlated with clinical PASI scores. By contrast, the levels of IL-8 were not correlated with the PASI score. The relationship between clinical severity and IL-8 concentration has been controversial in some previous studies [30,31]. In our study, IL-8 levels were highly sensitive in *S. pyogenes* induction, and some data appeared out of range in multiplex testing. Beyond that, our data showed no correlation with clinical PASI scores. Some biomarkers similar to IL-8, such as MCP-1, MIP-1α, and MIP-1β, were very sensitive to induction by *S. pyogenes*, independent of PASI results. IL-8, MCP-1, MIP-1α, and MIP-1β are essential for chemotaxis. In our opinion, variation in Th1 or Th17 upstream responses appears to correlate more with clinical PASI scores than cell chemotaxis.

In our subgrouping data, different markers appear to be present in different biologics. IFN-γ/IL-4 and IL-13 are important biomarkers in adalimumab. IFN-γ/IL-13 is an important one in ustekinumab. In different biologic mechanisms, they share the same biomarkers on IFN-γ and IL-13. Additionally, IL-17A seemed to be another marker for ustekinumab from our subgroup data. IFN-γ plays a pivotal role in the Th1 pathway. Adalimumab is a TNF-α inhibitor, a major cytokine in the Th1 pathway. It is reasonable that IFN-γ is an important marker of adalimumab. Ustekinumab blocks IL-12 and IL-23, which are upstream of Th17. It is logical that IL-17A is a better marker for ustekinumab than adalimumab. IFN-γ levels have a role in determining disease severity, as shown in a previous study [32]. The IL-17 levels of psoriasis patients were significantly higher than those of the controls and correlated to the severity of the disease [33]. IL-13 is an important cytokine of Th2, which plays important parts in both ustekinumab and adalimumab in our study. All of them are potentially important predictive biomarkers for guiding treatment selection. The lack of significance for ixekizumab, risankizumab, and guselkumab, respectively, may be due to the small sample size.

**Figure 3:**
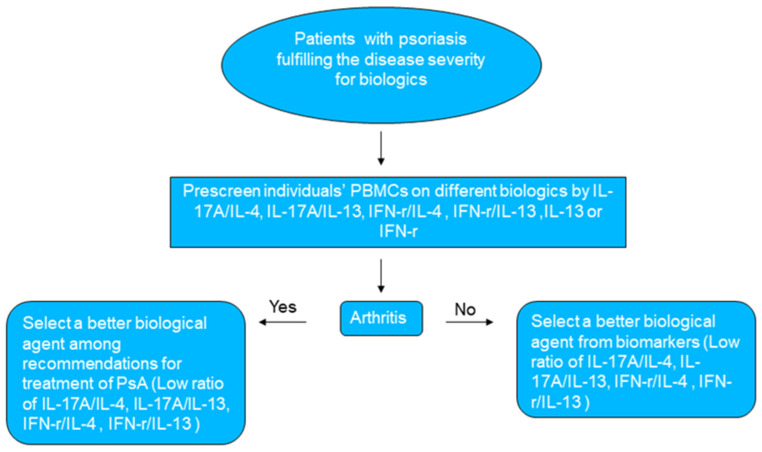
Suggested protocol for biologic selection before psoriasis treatment.

Appropriate biological therapy for individual patients with psoriasis can interrupt multiple inflammatory loops. TNF-α inhibitors could block dendritic cell activation, thereby inhibiting Th1-induced IFN-γ secretion and Th17-induced IL-17 secretion [34]. The exhausted phenotype of regulatory T cell dysfunction comprised reduced TGF-β release and increased IFN-γ production. Blocking IL-17 will reverse the exhausted phenotype of regulatory T cells to reverse the increased IFN-γ levels [35,36,37,38]. Furthermore, the induction of IL12p70 production on macrophages by IL-17 potentiates IFN-γ production by CD4+ cells. IL-17 inhibitor biologics could reduce IFN-γ production from macrophages and CD4+ cells [39]. IL-23, or IL-12/23, is the upstream cytokine of IL-17; the inhibitors of these share the same mechanism for blocking IFN-γ. In clinical data, we also observed suppression of serum IFN-γ levels with ustekinumab treatment in systemic lupus erythematosus patients [40]. The mechanisms of different biologics on IL-4 and IL-13 levels in individual patients with psoriasis remain unclear and need to be elucidated in the future. The role of immunity in Th1, Th17, and Th2 is frequently seen as a Yin and Yang [41]. Selecting a biologic that downregulates IFN-γ and IL-17A and upregulates IL-13 and IL-4 leads to better outcomes in individually tailored treatments. By prescreening patients, we could select a suitable biologic for an individual patient by following the flow chart in Figure 3. This study has some limitations. First, the results of the study must be validated in a larger group to assess the variability and validity of our findings. Second, some measurement errors can occur when the concentrations of IL-13 or IL-4 are extremely low, which may have caused the extremely large ratios of IL-17A to IL-13 and IFN-γ to IL-13 or IL-4 in some of our observations. More clinical research is necessary to address these limitations.

## 5. Conclusions

Psoriasis is a complex disease associated with genetics, environmental factors, and immune reactions, influencing diverse clinical characteristics and responses to treatment. We created an innovative, economic, and convenient platform to prescreen the efficacy of biological treatments for individual patients. The markers IFN-γ, IL-13, IFN-γ/IL13, IL-17A/IL-13, IFN-γ/IL4, and IL-17A/IL-4 are representative of predicting the efficacy of psoriasis treatment. These markers imply the importance of not only the Th1/Th17 pathway but also the Th2 pathway. Although the cohort analyzed was small, this study may be a valuable step toward predicting the responses of patients with psoriasis to a given therapy and, thus, toward individually tailored treatments.

## Figures and Tables

**Table 1:** Demographic and clinical characteristics of psoriasis patients (*n* = 20).

Pt	Age/ Gender	PsO/ PsA	Time of PBMC Test	Course and Order Sequence of Biologics	PASI Results (Consumption Time of Drugs on Cell Test)	Other Systemic Disease
A	52 y/M	+/−	10th month of adalimumab	6 months of ustekinumab 12 months of adalimumab	PASI 19 (6 months) PASI 100 (10 months)	-
B	43 y/M	+/+	2nd month of ustekinumab	6 months of adalimumab 6 months of ustekinumab	PASI-7 (6 months) PASI 99 (6 months)	Alcoholic hepatitis TAILS
C	44 y/M	+/−	5th month of adalimumab	6 months of ustekinumab 24 months of adalimumab	PASI 8 (6 months) PASI 96 (6 months)	-
D	59 y/M	+/+	12th month of adalimumab	12 months of adalimumab 18 months of ustekinumab *	PASI-100 (12 months) PASI 89 (6 months)	-
E	51 y/M	+/+	6th month of guselkumab	26 months of adalimumab 6 months of guselkumab 8 months of secukinumab *	PASI 52 (26 months) PASI 46 (6 months) PASI 85 (6 months)	-
F	55 y/F	+/+	29th month of golimumab	30 months of golimumab 18 months of adalimumab *	PASI 90 (29 months) PASI 80 (6 months)	-
G	44 y/M	+/+	20th month of secukinumab	54 months of ustekinumab 24 months of secukinumab 12 months of ixekizumab *	PASI 60 (54 months) PASI 89 (20 months) PASI 100 (6 months)	-
H	65 y/F	+/+	15th month of secukinumab	52 months of adalimumab 24 months of ustekinumab 24 months of secukinumab	PASI 26 (52 months) PASI 69 (24 months) PASI 93 (15 months)	-
I	57 y/F	+/+	1st month of secukinumab	47 months of adalimumab 15th month of secukinumab	PASI 47 (47 months) PASI 100 (6 months)	TAILS
J	40 y/M	+/−	1st month of ixekizumab	24 months of ustekinumab 12 months of ixekizumab	PASI 100 (24 months) PASI 92 (6 months)	-
K	54 y/M	+/−	1st month of ixekizumab	24 months of ustekinumab 6 months of ixekizumab	PASI 75 (24 months) PASI 100 (6 months)	HBV
L	42 y/M	+/−	2st week of ixekizumab	24 months of ustekinumab 6 months of ixekizumab	PASI 89 (24 months) PASI 94 (6 months)	-
M	65 y/F	+/−	18th month of secukinumab	27 months of adalimumab 28 months of secukinumab	PASI 74 (27 months) PASI 94 (18 months)	Tb
N	54 y/M	+/+	10th month of ixekizumab	24 months of ustekinumab 19 months of golimumab 12 months of ixekizumab	PASI 89 (24 months) PASI 66 (19 months) PASI 100 (10 months)	-
O	39 y/M	+/+	30th month of ustekinumab (2nd course of ustekinumab)	36 months of ustekinumab 6 months of secukinumab 30 months of ustekinumab	PASI 49 (6 months) PASI 84 (30 months in 2nd course)	Kikuchi Fujimoto disease MI
P	43 y/M	+/−	20th month of guselkumab	6 months of ixekizumab 24 months of guselkumab	PASI 100 (6 months) PASI 100 (20 months)	HTN CKD
Q	32 y/M	+/+	33th month of guselkumab	24 months of ustekinumab 33 months of guselkumab	PASI 92.5 (24 months) PASI 100 (33 months)	-
R	42 y/M	+/−	21th month of risankizumab	24 months of ustekinumab 21 months of risankizumab	PASI 100 (24 months) PASI 100 (21 months)	-
S	43 y/M	+/−	12th month of ixekizumab	24 months of ustekinumab 12 months of ixekizumab	PASI 42 (24 months) PASI 100 (12 months)	Hyperlipidemia
T	43 y/M	+/−	0th month of certolizumab	12 months of ustekinumab 12 months of risankizumab	PASI 80 (12 months) PASI 93 (12 months)	-

PsO = psoriasis; PsA = psoriatic arthritis; HBV = hepatitis B virus; TAILS = TNF-α inhibitor-induced systemic lupus erythematosus; Tb = tuberculosis; MI = myocardial infarction; CKD = chronic kidney disease. Patients received a PBMC test before biologics labeled with *. Patient O received a PBMC test in the 2nd course of ustekinumab.

**Table 2:** Laboratory profiles of patients with administered biologics (*n* = 20).

Pt	Before	After Bio	Induction	After Bio	Induction	After Bio	Induction	After Bio	Induction	After Bio	Induction	After Bio	Biologics
	Absolute PASI score		IFN-γ (pg/mL)		IL13 (pg/mL)		IFN-γ/IL13		IL4 (pg/mL)		IFN-γ/IL4		
A	3.2	2.6	13.72	12.43	4.19	3.41	3.27	3.46	0.09 0.09	0.2			ada
	3.2	0		11.97		3.55		3.36		OOR<			ust
B	15	16	74.46	88.28	1.24	0.81	62.47	108.99	5.54	3.56	13.44	24.8	ada
	15	0.2		71.02		1.24		57.27		5.45		13.03	ust
C	13	0.5	22.9	19.71	0.5	0.59	45.8	33.41	6.01	4.76	3.81	4.14	ada
	13	12		21.97		0.5		43.94		5.6		3.92	ust
D	7.5	15	200.39	222.11	5.19	4.31	38.61	51.53	13.94	13.64	14.38	16.28	ada
	7.5	0.8		201.66		5.28		38.19		14.48		13.93	ust
E	25	12	92.6	76.99	1.53	1.53	60.52	50.32	12.17	9.41	7.61	8.18	ada
	8	4.3		85.17		1.34		63.56		12.34		6.9	gus
	8	1.2		80.12		1.71		46.85		12.94		6.19	sec
F	4.1	0.8	107.72	105.21	2.99	2.22	33.69	47.39	10.94	8.62	9.85	12.2	ada
	4.1	0.4		107.68		2.22		48.5		8.72		12.35	gol
G	31.2	0	100.13	76.76	8.44	5.69	11.86	13.49	11.74	11.01	8.53	6.97	ixe
	56.5	22.4		102.31		6.73		15.2		11.44		8.94	ust
	22.4	2.4		84.74		7.78		10.89		11.49		7.38	sec
H	11.4	3.5	85.68	89.51	2.74	2.91	31.27	30.76	11.35	11.43	7.55	7.83	ust
	15.5	11.4		102.91		2.04		50.45		9.76		10.54	ada
	15.3	1		66.92		2.57		26.35		11.23		5.96	sec
I	3.8	2	244.94	290.14	3.05	2.73	80.31	106.28	10.73	10.82	22.83	26.82	ada
	2	0		269.05		4.27		63.01		13.1		20.54	sec
J	15	1.2	273.76	297.09	2.15	2.24	127.33	132.63	11.74	12.17	23.32	24.41	ixe
	16	0		268.46		2.24		119.85		12.02		22.33	ust
K	15	3.7	367.78	365.71	6.14	5.86	59.9	62.41	13.51	13.31	27.22	27.48	ust
	11.7	0		383.17		6.27		61.11		13.81		27.75	ixe
L	36.8	4	194.87	134.85	1.98	1.62	98.41	83.24	14.22	11.55	13.7	11.68	ust
	16	1		81.47		1.44		56.57		9.97		8.17	ixe
M	20	5.2	247.16	262.11	1.71	2.06	144.54	127.24	7.77	9.77	31.81	26.83	ada
	16	0.9		297.59		3.9		76.3		15.15		19.64	sec
N	27	3.1	66.37	61.69	1.76	1.76	37.71	35.2	3.82	3.53	17.37	17.65	ust
		9.1		66.72		1.45		46.01		3.46		19.28	gol
		0		63.72		1.86		34.26		3.85		16.55	ixe
O	19.2	3	249.87	194.12	1.44	0.84	173.52	231.1	14.67	12.02	17.03	16.15	ust
		9.8		188.89		1.25		151.11		11.7		16.14	sec
P	13.1	0	50	53.26	1.66	1.66	30.12	32.08	3.23	3.41	15.48	15.62	ixe
		0		40.67		1.86		21.87		3.01		13.51	gus
Q	16	1.2	32.1	40	0.16	0.59	200.6	71.3	2.35	2.44	13.7	17.2	ust
	27	0		24.2		0.24		100.8		1.88		12.9	gus
R	26	0	52.6	49.8	0.32	0.32	164.3	104.3	2.48	1.56	21.2	31.3	ust
	16.4	0		31.2		0.46		97.5		1.41		22.1	ris
S	7.8	4.5	29	27.64	0.34	0.2	85.3	84	4.06	3.95	7.14	7.21	ust
	8.5	0		28.5		0.34		138.2		3.96		7	ixe
T	18.4	3.6	41.02	31.78	0.42	0.2	97.67	158.9	2.01	2.42	20.4	13.1	ust
		1.2		27.98		0.56		49.96		2		13.99	ris

Pt = patient; PASI = psoriasis area and severity index; OOR< = out of range below; Bio = biologics; ada = adalimumab; ust = ustekinumab; gus = guselkumab; ris = risankizumab; gol = golimumab; ixe = ixekizumab; sec = secukinumab.

**Table 3:** Clinical association between clinical PASI and laboratory profile by reduction rate and difference.

Reduction	PASI		IFN-r		IL-13		IL-4		IL17A	
Ratio	r_s_	*p* Value	r_s_	*p* Value	r_s_	*p* Value	r_s_	*p* Value	r_s_	*p* Value
PASI	1.00		0.22	0.152	−0.48	0.001 **	−0.05	0.753	0.12	0.444
IFN-r	0.22	0.152	1.00		0.18	0.255	0.40	0.007 **	0.49	0.001 **
IL-13	−0.48	0.001 **	0.18	0.255	1.00		0.33	0.030 *	0.21	0.161
IL-4	−0.05	0.753	0.40	0.007 **	0.33	0.030 *	1.00		0.74	<0.001 **
IFN-r/IL13	0.52	<0.001 **	0.35	0.022 *	−0.76	<0.001 **	0.01	0.958	0.10	0.516
IFN-r/IL4	0.29	0.061	0.51	0.001 **	−0.09	0.581	−0.40	0.009 **	−0.13	0.394
IL17A	0.12	0.444	0.49	0.001 **	0.21	0.161	0.74	<0.001 **	1.00	
IL17A/IL13	0.47	0.001 **	0.27	0.081	−0.60	<0.001 **	0.25	0.108	0.51	<0.001 **
IL17A/IL4	0.36	0.019 *	0.54	<0.001 **	0.02	0.914	0.27	0.087	0.71	<0.001 **
IL-6	0.04	0.805	0.66	<0.001 **	0.22	0.145	0.52	<0.001 **	0.51	<0.001 **
Reduction	PASI		IFN-r		IL-13		IL-4		IL-17A	
Difference	r_s_	*p* value	r_s_	*p* value	r_s_	*p* value	r_s_	*p* value	r_s_	*p* value
PASI	1.00		0.45	0.002 **	−0.14	0.366	0.13	0.392	0.10	0.512
IFN-r	0.45	0.002 **	1.00		0.18	0.251	0.49	0.001 **	0.48	0.001 **
IL-13	−0.14	0.366	0.18	0.251	1.00		0.57	<0.001 **	0.41	0.006 **
IL-4	0.13	0.392	0.49	0.001 **	0.57	<0.001 **	1.00		0.77	<0.001 **
IFN-r/IL13	0.41	0.006 **	0.30	0.051	−0.62	<0.001 **	−0.04	0.780	−0.05	0.753
IFN-r/IL4	0.32	0.041 *	0.44	0.004 **	−0.25	0.115	−0.29	0.063	−0.21	0.191
IL17A	0.10	0.512	0.48	0.001 **	0.41	0.006 **	0.77	<0.001 **	1.00	
IL17A/IL13	0.33	0.029 *	0.32	0.032 *	−0.48	0.001 **	0.16	0.295	0.25	0.098
IL17A/IL4	0.39	0.011 *	0.49	0.001 **	−0.02	0.894	0.15	0.347	0.47	0.002 **
IL-6	0.2	0.195	0.58	<0.001 **	0.25	0.104	0.53	<0.001 **	0.4	0.007 **

Spearman’s rho. * *p* < 0.05, ** *p* < 0.01.

**Table 4:** Correlation between clinical PASI and biomarkers in PsO only and PsO + PsA groups.

PsO Only (*n* = 10)						PsO + PsA (*n* = 10)					
	PASI			PASI			PASI			PASI	
Ratio	r_s_	*p* Value	Difference	r_s_	*p* Value	Ratio	r_s_	*p* Value	Difference	r_s_	*p* Value
PASI	1		PASI	1		PASI	1		PASI	1	
IFN-r	0.02	0.935	IFN-r	0.46	0.040 *	IFN-r	0.35	0.093	IFN-r	0.53	0.008 **
IL-13	−0.35	0.128	IL-13	−0.4	0.079	IL-13	−0.46	0.023 *	IL-13	−0.03	0.88
IL-4	0.27	0.257	IL-4	0.1	0.678	IL-4	−0.31	0.147	IL-4	0.11	0.598
IFN-r/IL13	0.3	0.199	IFN-r/IL13	0.63	0.003 **	IFN-r/IL13	0.56	0.005 **	IFN-r/IL13	0.35	0.094
IFN-r/IL4	−0.26	0.297	IFN-r/IL4	0.23	0.369	IFN-r/IL4	0.5	0.012 *	IFN-r/IL4	0.38	0.066
IL17A	0.27	0.241	IL17A	0.12	0.609	IL17A	−0.14	0.525	IL17A	0.09	0.661
IL17A/IL13	0.47	0.038 *	IL17A/IL13	0.35	0.132	IL17A/IL13	0.32	0.124	IL17A/IL13	0.43	0.036 *
IL17A/IL4	0.36	0.137	IL17A/IL4	0.27	0.278	IL17A/IL4	0.25	0.238	IL17A/IL4	0.56	0.005 **
IL-6	0.05	0.833	IL-6	0.05	0.843	IL-6	0.1	0.627	IL-6	0.34	0.106

Spearman’s rho. * *p* < 0.05, ** *p* < 0.01.

## Data Availability

The data presented in this study are available on request from the corresponding author due to the privacy of research participants.
